# Contribution of researchers in the Arab region to peer-reviewed literature on mental health and well-being of university students

**DOI:** 10.1186/s13033-021-00477-9

**Published:** 2021-05-26

**Authors:** Waleed M. Sweileh

**Affiliations:** Department of Physiology, Pharmacology/Toxicology, Division of Biomedical Sciences, College of Medicine and Health Sciences, An-Najah National University, Nablus, Palestine

**Keywords:** Mental health, Well-being, University students, Arab region, Research output

## Abstract

**Objective:**

The current study aimed at investigating the contribution of researchers in the Arab region to the field of mental health and well-being of university students using bibliometric tools.

**Method:**

Relevant literature was obtained from the Scopus database for the period from 2001–2020. Examples of keywords used in the query included “college student”, “university student”, and undergraduate student” combined with keywords such as wellbeing, wellness, suicide, and anxiety. No language restriction was used. Only research articles were considered. The search query was validated. Bibliometric indicators and mappings such as active countries, institutions, authors, highly cited documents, and the most frequently encountered topics were identified and discussed to shed light on research gaps in the Arab region. Research gaps were also identified. The analysis was carried out on February 12, 2021.

**Results:**

The search query returned 309 research articles published by authors from 17 different Arab countries. Less than one-third (n = 97, 31.4%) of the retrieved articles were carried out in collaboration with authors from 39 non-Arab countries, mainly from the United Kingdom and the United States. The overall contribution of researchers from the Arab region to global research in the field was 5.6%. In total, 1212 authors from 791 different institutions participated in publishing the retrieved research articles. At the country level, Saudi Arabia (n = 125, 40.5%) ranked first, followed by Jordan, Egypt, and Lebanon. At the institutional level, *The University of Jordan* (n = 25, 8.1%) ranked first, followed by *King Saud University*, and *Kuwait University*. The retrieved articles included 132 (42.7%) articles on stress/distress, 95 (30.7%) on anxiety, 61 (19.7%) on depression. Knowledge gaps on suicide, eating disorders, substance use, and happiness were identified. The retrieved articles appeared in 193 different journals and approximately two-thirds of the active journal were in general medicine, public health, and education.

**Conclusions:**

The contribution of researchers in the Arab region to the field showed a noticeable increase with time. However, important research gaps were identified. The contribution was confined to authors from a limited number of Arab countries. Funding and international research collaboration for the mental health and well-being of students need to be strengthened.

## Background

The Arab youth (aged 15–24 years) constitutes approximately 100 million of the total population of the 22 Arab countries (423 million) [1]. In the past three decades, there has been a tremendous change in the number and type of higher education institutions in the Arab region to meet the demands of increasing numbers of university students [2]. University students in the Arab region represent a sizable number of young population that requires special policies in education, employment, and health. Achieving the United Nations’ Sustainable Development Goals (SDGs) requires investment in the health of university students to ensure future healthy adult generations.

Mental health is an important component of physical health and is defined as “*a state of well-being in which an individual realizes his or her own abilities, can cope with the normal stresses of life, can work productively and is able to make a contribution to his or her community*” [3]. In 2013, all Member States of the World Health Assembly approved and adopted the *“Comprehensive Mental Health Action Plan for 2013–2020”* to promote mental health nationally and globally [4]. University students in the Arab region are exposed to different types of academic and non-academic stressors that might be different from their peers in Europe, North America, and other world regions [5–7]. The Arab region has witnessed major events in the past three decades such as the Iraqi war, Arab Spring protests, the political unrest in Syria, Yemen, Egypt, Sudan, Palestine, Lebanon, Somalia, and Tunisia, the massive numbers of refugees, the Arab-Israeli conflict, the diplomatic crisis in the Arab Gulf, the violent Islamic groups, and finally the COVID-19 pandemic.

Students in the Arab region suffer many life pressures that are unique to the Arab region. For example, in most Arab countries there is a lack of governmental and non-governmental bodies to support student loans with no or minimum financial interest [8, 9]. Students in the Arab region are also pressured by mass unemployment, minimum decent job opportunities, and an uncertain future [10]. Family income in most Arab countries is usually low which increases the pressure on young adults to succeed academically to save money for their large families [11, 12]. The vast growth of technology and its use in academic life put further psychological pressures on students with limited income. In some Arab countries, universities are located in major cities which creates another psychological pressure on students from the suburbs and villages due to lack of efficient transportation and decent on-campus housing [13]. Finally, the stress of war, political instability, poor economy, uncertain future, and sometimes the lack of democracy are major contributing factors to mental health problems among university/college students in the Arab region [10, 14, 15]. Globalization has influenced the young people generation in the Arab region in a way that made some of the hopeless young people travel through the sea looking for a better and safer life. Students in the Arab region can easily compare the lifestyle and life opportunities of students in other parts of the world with their living conditions [16, 17]. For example, in Arab countries, politics, religion, and culture impose a lot of constraints on students’ behavior in contrast to those in developed countries who have a larger margin of democracy and freedom including gender equality [18, 19]. For all the reasons mentioned above, the mental health status and well-being of university students in the Arab region deserve special attention and investigation.

Assessing research publications on mental health and well-being of university students in the Arab region is the first initial step in changing the future health of young generations. At the global level, a study was recently published and assessed the worldwide research publications on mental health and well-being of university students [20]. However, none was carried out at the Arab regional level. Based on the information presented above, the current study was a bibliometric analysis carried out to achieve the following objectives:

1. Determine the volume and growth of research publications on mental health and well-being of university students in the Arab region and compare it with global research in the same field.
2. Determine the hot spots and gaps in research on mental health and well-being of university students in the Arab region.
3. Determine the most active countries, institutions, and authors involved in this field at the regional level.

Achieving the above-mentioned objectives will add up to the existing literature on Arab youth and will enrich the field of mental health.

## Method

The current study, including the analysis, was carried out in February 2021. The author relied on the Scopus database to retrieve relevant documents because (1) Scopus is available to the author through the “Research4life” initiative, (2) Scopus is the largest database available, (3) and it has indexed journals in the field of health, social sciences, life sciences, and physical sciences [21, 22]. Scopus has been previously used to carry out analysis and visualization of research publications in different health fields [23–25]. Despite that Scopus is one of the largest databases, it has certain drawbacks. In the Arab region, there are many general medical journals. Most medical journals in the Arab Gulf countries such as *Annals of Saudi Medicine*, *Saudi Medical Journal*, *Medical Principles and Practice*, *Emirates Medical Journal, and Omani Medical Journal* are indexed in Scopus and have a moderate impact factor. Jordan, Egypt, Iraq, and Tunisia have several Scopus-indexed medical journals such as *Jordan Medical Journal*, *Eastern Mediterranean Health Journal*, *New Iraqi Journal of Medicine, Tunisie Medicale*, *Journal Medical Libanais*, and *Egyptian Journal of Public Health.* In the field of mental health, the *Egyptian Journal of Neurology, Psychiatry, and Neurosurgery* and the *Middle East Current Psychiatry* journals are indexed in Scopus. Therefore, articles published in regional medical journals were mostly retrieved and analyzed and the number of missing data is expected to be insignificant.

The author utilized the keywords relevant to university students, mental health, and well-being present in the previously published bibliometric study [20]. These keywords included university or college or “higher education” or “tertiary education” or “post-secondary education” or “undergrad* student” or “grad∗ student” or “master’s student” or “doctoral student” or “Ph.D. student”. These keywords must be associated with the keyword “student” in the title of the article. Keywords related to mental health and well-being included: (“mental illness” or “mental disorder*” or “mental distress” or “psychological distress” or “psychopathology” or “depression” or “anxiety” or “stress” or “suicide” or “eating disorder*” or “substance use”) or (“well-being” or “wellbeing” or “wellness” or “life satisfaction” or “happiness” or “positive affect” or “purpose in life” or “personal growth” or “self-determination”). The search query included the list of all Arab countries in the country affiliation to limit the retrieved documents to those in which at least one author was from one of the Arab countries.

In the current study, the author made the study period from January 01, 2001, to December 31, 2020, for the following two reasons: (1) applying the search query in Scopus showed that a very limited number of articles (only eight articles) were published before the year 2001, and (2) the assigned study period covers the introduction of Millennium Development Goals and SDGs which emphasized the importance of both mental health and adolescent health [26–28]. The types of documents included in the analysis were only research articles published in peer-reviewed journals. Other types were excluded. No restrictions were made regarding the language of the article. Therefore, English and non-English research articles were included in the analysis. All non-English articles in the Scopus have an English title and abstract which facilitated the process of validation and analysis. In the current study, an article on the psychometric properties of the Beck depression scale [29] was excluded because it did not focus on the epidemiology of mental health and well-being of the students. The final search query was entered in the Scopus and the retrieved research articles were validated by reading the title and abstract of the top 100 cited research articles to make sure that all retrieved articles fit the criteria of the study: (1) carried out by at least one or more authors from the Arab states, and (2) carried out on university students. The result of the validation of the finalized search query was optimum with no false-positive results.

The current study was a quantitative bibliometric analysis and visualization of research on mental health and well-being of students in the Arab region. Bibliometric analysis was carried out using the “analyze” function in Scopus which provides detailed information about annual growth, list of involved countries, institutions, and authors. Scopus database allows for the export of results into Microsoft Excel as a “csv” file for tabulation and drawing of the results. Scopus also allows for the export of all data about the retrieved articles into a csv file to be used in mapping programs such as VOSviewer [30]. VOSviewer is a free online program that is commonly used for bibliometric mapping such as visualization of most frequently encountered author keywords. In the current study, citations for the retrieved articles were exported to Microsoft Excel using the function “citation analysis” in Scopus. The value of the Hirsch-index (H-index) was used to assess the impact of countries, journals, authors, and articles.

One of the objectives of the current study was to assess the contribution of researchers in the Arab region relative to the global research activity in the field of mental health and well-being of university students. To achieve this, the final search query was implemented twice, once with all Arab countries listed in the search query and once without any specific country listed in the query to retrieve the global research articles in this field.

## Results

**Fig. 1:**
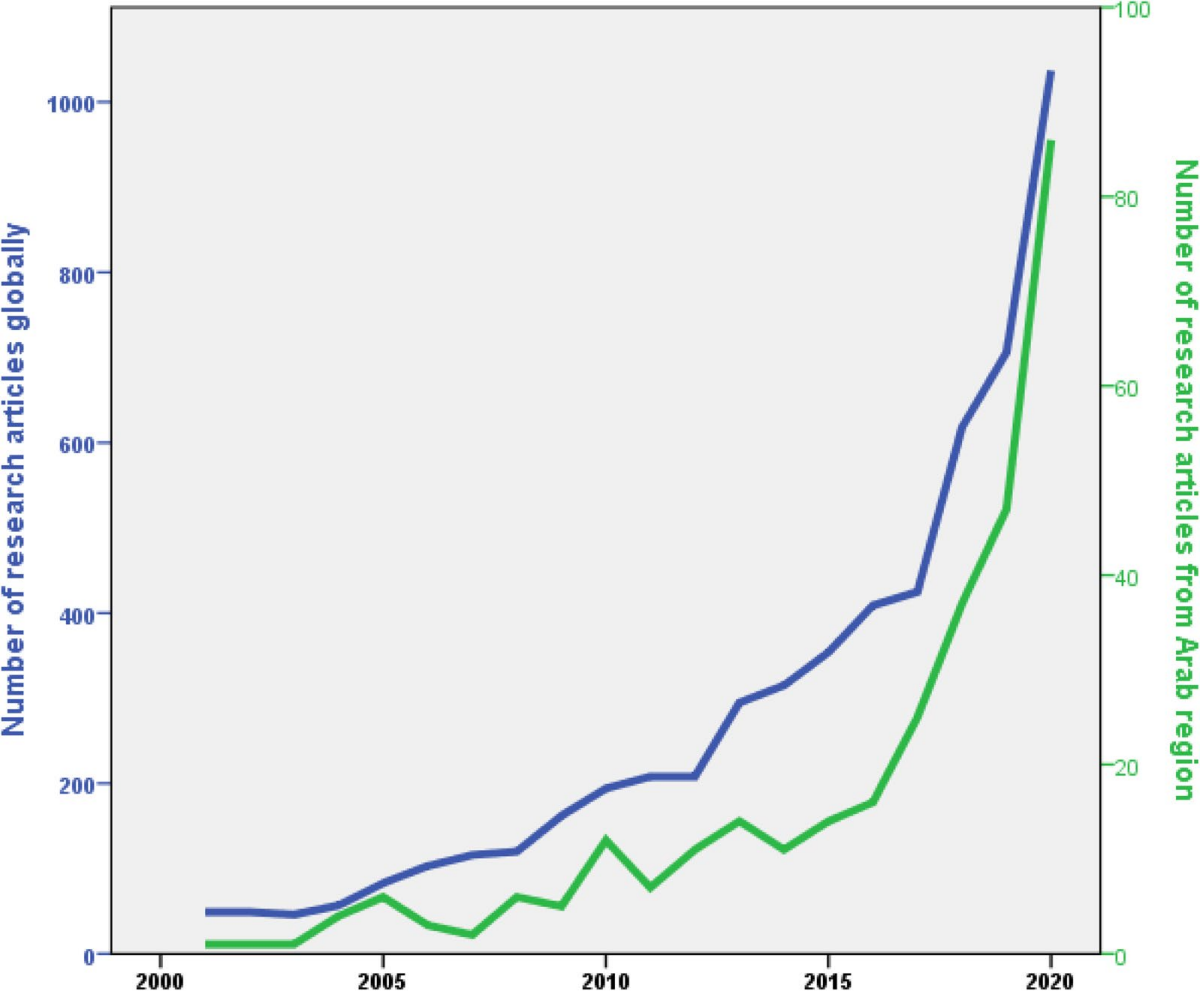
Annual growth of research publications on mental health and well-being of students by researchers in the Arab region

**Fig. 2:**
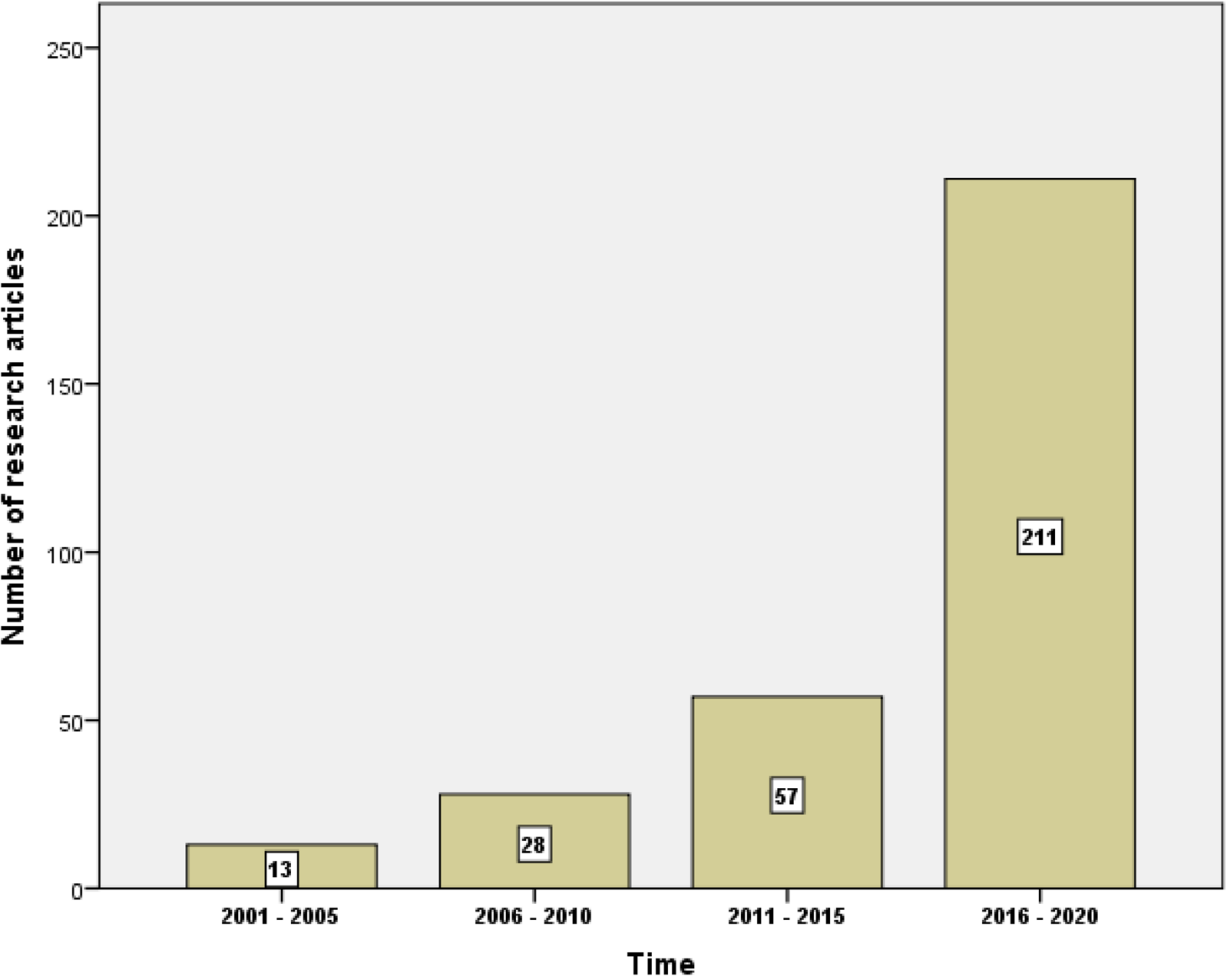
Number of research publications per 5-year interval by researchers in the Arab region on the mental health and wellbeing of students. There was a significant (p < 0.001) increase observed in the last time interval relative to the interval before

The search query found 309 research articles while the global research output in the field was 5554. Therefore, the research contribution of researchers in the Arab region represents 5.6% of the global research on mental health and well-being of university students. Figure 1 shows a significant increase in the number of publications from the Arab region with time while Fig. 2 compares the annual growth of publications from the Arab region and globally. The figure shows an increase in the contribution of researchers from the Arab region relative to global research output with time.

Table 1 shows the list of Arab countries with their research output, Saudi Arabia (n = 125, 40.5%) ranked first followed by Jordan (n = 57, 18.4%), Egypt (n = 45, 14.6%), and Lebanon (n = 28, 9.1%). Out of the 22 Arab countries, 17 contributed to the retrieved articles while the remaining (Djibouti, Mauritania, Somalia, Algeria, and Comoros) had no research activity in this field. The Arab countries collaborated with 39 non-Arab countries to publish the retrieved articles. A total of 212 (68.6%) articles were produced without international research collaboration and 97 (31.4%) articles were produced with international research collaboration. The UK (n = 23, 7.4%) was the major collaborating country followed by the USA, Pakistan, Australia, and Malaysia.

**Table 1:** List of Arab countries and the contribution of authors in each country to the mental health and well-being research on university students

Country	Number of publications	% (N = 309)
Saudi Arabia	125	40.5
Jordan	57	18.4
Egypt	45	14.6
Lebanon	28	9.1
Kuwait	26	8.4
United Arab Emirates	18	5.8
Iraq	13	4.2
Tunisia	10	3.2
Palestine	8	2.6
Oman	7	2.3
Bahrain	5	1.6
Qatar	5	1.6
Syrian Arab Republic	5	1.6
Yemen	5	1.6
Morocco	3	1.0
Sudan	3	1.0
Libyan Arab Jamahiriya	1	0.3
Algeria, Mauritania, Somalia, Djibouti, Comoros	0	0.0

In total, 1212 authors, an average of 3.9 authors per article. There were 43 (13.9%) single-authored articles, 72 (23.3%) two-authored articles, 52 (16.8%) three-authored articles, 34 (11.0%) four-authored articles while the remaining 108 (35.0%) were multi-authored (≥ 5) articles and the remaining were multi-authored. Abdel-Khalek, A.M. (Alexandria University, Department of Psychology) ranked first with 15 (5.2%) articles. Hamdan-Mansour, A.M.'s (The University of Jordan, Department of Community Health Nursing) ranked second with 7 articles.

Authors from 791 different institutions participated in publishing the retrieved articles. Table 2 shows the top ten active institutions in the field. The University of Jordan ranked first with 25 (8.0%) publications followed by King Saud University (n = 22, 7.1%), Kuwait University (n = 19, 6.1%), and Imam Abdulrahman Bin Faisal University (n = 17, 5.5%). At the global level, The University of Jordan ranked 14th while King Saud University ranked 25th in this field.

**Table 2:** Top ten active institutions in Arab countries in publishing articles on mental health and well-being of university students

Rank	Institutions	Number	% (N = 309)	Country
1	*The University of Jordan*	25	8.1	Jordan
2	*King Saud University*	22	7.1	Saudi Arabia
3	*Kuwait University*	19	6.1	Kuwait
4	*Imam Abdulrahman Bin Faisal university*	17	5.5	Saudi Arabia
5	*Jordan University of Science and Technology*	13	4.2	Jordan
6	*King Abdulaziz University*	12	3.9	Saudi Arabia
7	*Assiut University*	8	2.6	Egypt
7	*Umm Al Qura University*	8	2.6	Saudi Arabia
7	*Alexandria University*	8	2.6	Egypt
10	*An-Najah National University*	7	2.3	Palestine
10	*King Saud bin Abdulaziz University for Health Sciences*	7	2.3	Saudi Arabia
10	*Princess Nourah bint Abdulrahman University*	7	2.3	Saudi Arabia

The retrieved articles were published in 193 different journals led by the *Psychological Report* journal (n = 9, 2.9%) and the *Journal of Taibah University Medical Sciences* (n = 9, 2.9%). In total, 26 (13.5%) different journals published three or more research articles with a total of 110 (35.9%). Table 3 shows the 26 journals with ≥ 3 articles. Seven out of the 26 journals were based in the Arab region. The majority of the journals in the list were in the general medical field or public health. Out of the 26 journals, 16 (64.0%) were in general medicine, health, and education while the remaining were in psychiatry/mental health/psychology.

**Table 3:** list of journals that published a minimum contribution of three research articles by researchers in the Arab region on the mental health and well-being of university students

Journal name	Number	% (N = 309)	h-index	Scope	Country/institution affiliation (Yes, No)
*Journal Of Taibah University Medical Sciences*	9	2.9	14	General medicine	Yes
*Psychological Reports*	9	2.9	63	Psychology	No
*Indian Journal Of Public Health Research And Development*	6	1.9	11	Public health	No
*Journal Of Psychosocial Nursing And Mental Health Services*	6	1.9	33	Mental health nursing/ Psychiatry	No
*Saudi Medical Journal*	6	1.9	48	General Medicine	Yes
*Middle East Current Psychiatry*	5	1.6	6	Psychiatry/ Mental health	Yes
*Perspectives In Psychiatric Care*	5	1.6	32	Psychiatric mental health	No
*Plos One*	5	1.6	300	General/ Multidisciplinary	No
*Social Behavior And Personality*	5	1.6	53	Social Psychology	No
*BMC Medical Education*	4	1.3	61	**Social Sciences/ Education**	No
*BMC Psychiatry*	4	1.3	88	Psychiatry/ Mental health	No
*Central European Journal Of Public Health*	4	1.3	32	Public Health	No
*Eastern Mediterranean Health Journal*	4	1.3	46	General medicine	Yes
*International Review Of Psychiatry*	4	1.3	78	Psychiatry/ Mental health	No
*Neuropsychiatric Disease And Treatment*	4	1.3	62	Psychiatry and Neuroscience	No
*Academic Psychiatry*	3	1.0	40	Psychiatry/ Education	No
*Asian EFL Journal*	3	1.0	10	**Social Science/ Education**	No
*Dirasat Human And Social Sciences*	3	1.0	3	**Social sciences**	Yes
*International Journal Of Adolescence And Youth*	3	1.0	16	**Social sciences/ Health**	No
*International Journal Of Preventive Medicine*	3	1.0	35	General medicine/ Public health	No
*Jordan Medical Journal*	3	1.0	6	General medicine	No
*Journal Of The Social Sciences*	3	1.0	5	Social Sciences	Yes
*Mental Health Religion And Culture*	3	1.0	40	Psychiatry/Mental health/ Psychology	No
*Open Access Macedonian Journal Of Medical Sciences*	3	1.0	13	General Medicine	No
*Tunisie Medicale*	3	1.0	17	General medicine	Yes

The retrieved articles received 3443 citations, an average of 11.1, and H-index of 34. Table 4 shows the top ten articles that received the highest citations. Four of the top-cited articles focused on the mental health and well-being of medical/nursing students. Seven of the top-cited articles were published in journals unrelated to psychiatry/psychology. The top-cited article received 137 citations and focused on the impact of stress on medical students in medical colleges in Saudi Arabia.

**Table 4:** Top ten cited research articles on mental health and well-being of university students from Arab region

Rank	Title	Year	Journals	Number of citations
1	*“Stress and its effects on medical students: A cross-sectional study at a college of medicine in Saudi Arabia”*	2011	*Journal of Health, Population and Nutrition*	136
2	*“Depression, anxiety, and smartphone addiction in university students- A cross sectional study”*	2017	*PLoS ONE*	104
3	*“Internet addiction and relationships with insomnia, anxiety, depression, stress and self-esteem in university students: A cross-sectional designed study”*	2016	*PLoS ONE*	89
3	*“Cognitive emotions: Depression and anxiety in medical students and staff”*	2009	*Journal of Critical Care*	89
5	*“Quality of life, subjective well-being, and religiosity in Muslim college students”*	2010	*Quality of Life Research*	82
6	*“Humour styles, personality, and well-being among Lebanese university students”*	2004	*European Journal of Personality*	79
6	*“Undergraduate nursing students’ stress sources and coping behaviours during their initial period of clinical training: A Jordanian perspective”*	2012	*Nurse Education in Practice*	76
8	*“The Relations Among Social Media Addiction, Self-Esteem, and Life Satisfaction in University Students”*	2017	*Social Science Computer Review*	69
9	*“Stress, anxiety, and depression among medical students in a multiethnic setting”*	2015	*Neuropsychiatric Disease and Treatment*	66
10	*“Middle East Respiratory Syndrome-Corona Virus (MERS-CoV) associated stress among medical students at a university teaching hospital in Saudi Arabia”*	2020	*Journal of Infection and Public Health*	65

**Fig. 3:**
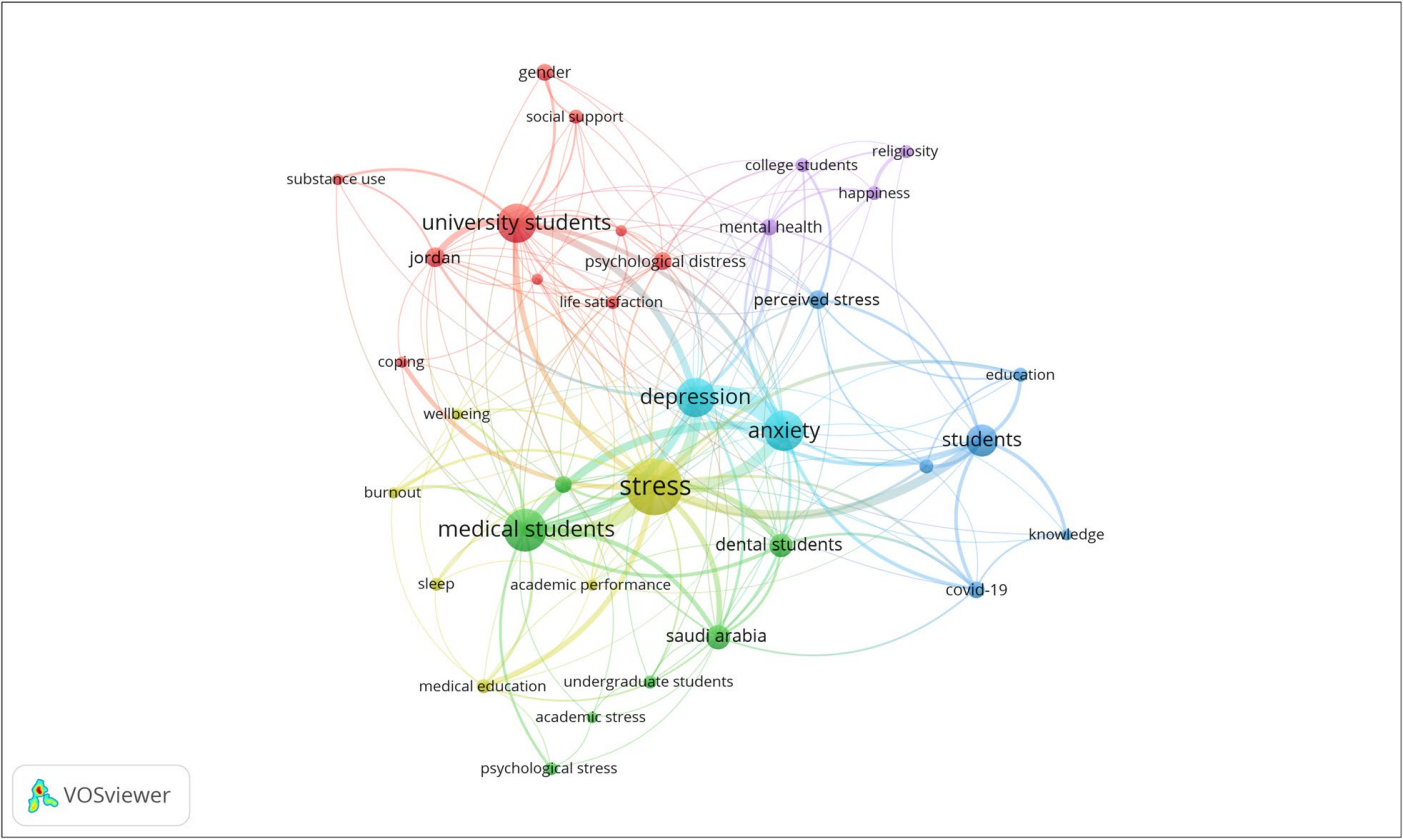
Network visualization map of author keywords (n = 35) with a minimum occurrence of 5 times

Figure 3 shows the network visualization of author keywords with a minimum of 5 occurrences. The map included 35 author keywords from a total of 650 author keywords in the retrieved literature. Keywords with the largest circle size were the most frequent and included stress, depression, anxiety, substance use, life satisfaction, happiness, well-being, burnout, sleep, and self-esteem. The map indicated that the most recent literature was mental health-related publications among university students during the COVID-19 pandemic.

Analysis of the retrieved articles indicated that 132 (42.7%) were about stress/distress, 95 (30.7%) about anxiety, 61 (19.7%) articles about depression, 33 (10.7%) about well-being, 21 (6.8%) about substance use, 11 (3.6%) about eating disorders, 8 (2.6%) about sleep disorders, 6 (1.9%) about happiness, 6 (1.9%) about suicide, 5 (1.9%) about life satisfaction, 5 (1.9%) about burnout, 3 (1.0%) about self-esteem, and 12 (3.9%) about the mental health in general. There was a certain overlap which made the total percentage above 100%.

Table 5 shows that the retrieved articles belonged to 10 different subject areas with the subject area of medicine constituting the major one (n = 164) followed by social sciences, psychology, nursing, humanities, dentistry, biochemistry, pharmacology, neuroscience, and multidisciplinary subjects. For global research publications, the subject areas were medicine, psychology, social sciences, nursing, humanities, biochemistry, neuroscience, pharmacology, and health professions.

**Table 5:** Top five subject areas of the retrieved research articles produced by the Arab region and globally

Subject area (Arab region)	Number	% (N = 309)	Subject area (Global)	Number	% (N = 5554)
Medicine	164	53.1	Medicine	2517	45.3
Social Sciences	59	19.1	Psychology	1828	32.9
Psychology	56	18.1	Social Sciences	1724	31.0
Nursing	35	11.3	Nursing	473	8.5
Arts and Humanities	20	6.5	Arts and Humanities	353	6.4

## Discussion

The current study investigated and assessed the contribution of researchers from Arab countries to the literature on mental health and well-being of university students. The study showed an increasing contribution with time. However, the overall contribution was lesser than expected given the life stressors and the growing numbers of the youth population in the Arab region. The results of the current study are in agreement with a previously published study about mental health research in the Arab region in which the authors concluded that research output in the Arab world has increased by almost 160% in the past ten years but the number of articles per capita remains remarkably lower for the Arab world compared to the rest of the world [31]. The contribution of Arab countries was confined to few Arab countries and the international research collaboration in this field was inadequate. Research publications from the Arab region had a higher percentage of articles in the field of nursing but a lesser percentage of articles in the field of medicine, psychology, and social sciences relative to global research publications. This indicates the strong research capacity of Arab researchers in the field of mental health nursing in certain institutions in Arab countries.

The current study showed that stress, depression, and anxiety were the main focus of mental health research on university students in the Arab region. These three topics constituted approximately 93% of the retrieved articles while other important topics were under-researched. Research on topics related to the well-being of students such as happiness, suicide, substance use disorders, eating disorders, and life satisfaction was under-researched. Similar findings were obtained by other researchers. A bibliometric study on substance use disorder in Arab countries indicated that this field is generally neglected and more efforts are needed to provide research data on this topic [32]. Studies on substance use among youth in Arab countries indicated that such a problem does exist among adolescents and youth [33–37]. To strengthen research on substance use disorders in Arab countries, the topic should be one of the top research priorities for funding given that substance use is included in the SDG 03 to be achieved by 2030. Another important research gap in Arab countries was eating disorders, especially among female students. Globalization through digital media has changed the perception of beauty criteria among young females in Arab countries [38]. Losing weight and maintaining a body shape similar to those in Hollywood became a real concern to young females. Very few studies have been carried out to assess disordered eating patterns among university females in the Arab world despite the expectation of a higher prevalence of eating disorders [39, 40]. Research on eating disorders is important since early detection and intervention through awareness and psychological support increases the chances of prevention and treatment [41, 42]. Research on happiness and life satisfaction among youth in Arab countries were also limited. The available studies on happiness among university students were carried out among young adults in the oil-rich Arab Gulf countries [43–47]. One potential reason for the limited number of research on this topic is the Islamic religious principles that prohibit pessimism and suicide. A study in Arab college students found that students who see themselves as religious have greater levels of mental health and happiness [48]. Similar findings were obtained in studies among university students in Turkey [49], Egypt, and Kuwait [50]. The argument about research on happiness applies to research on suicide among university students. In the current study, there was a very limited number of publications on suicide. Similar findings were reported by other authors regarding the limited research on suicide among Arab adolescents [51]. Despite low rates of suicide in Muslim communities, research on this topic should be encouraged to get more data to implement necessary interventions before the rates reach unmanageable levels [52–55]. It is expected that the armed conflict in different Arab countries, the massive numbers of displaced people, and the disruption of education and social life will create psychological traumas that could lead to higher rates of depression and suicidal ideation. University students in Arab countries with armed conflicts need to be screened for adjustment disorders. However, none, or very few publications were published from researchers in the Arab region on this regard. The under-researched topics need to be strengthened and supported. In general, researchers in the field of mental heal, psychology, and psychiatry need training on different types of research and on how to research in this field. Furthermore, establishing Institutional review boards to monitor the ethics of research on this human-related topic is needed in all countries and universities to facilitate research and promote publishability of the projects. It is also important that policy makers help researchers in implementing their research findings so that people feel a positive change produced by research in this field. Most reputable journals in the field of psychiatry and mental health have article processing charges and therefore supporting researchers financially will help to promote research publication to an international audience. Publishing in reputable journals can also create opportunities for international research collaboration which increases research output and enhance the quality of research publications. Such recommendations and actions to be taken to promote mental health research in general in the Arab region [55].

In the current study, authors from Saudi Arabia, Jordan, and Egypt dominated the retrieved literature. The leading role of Saudi Arabia in the Arab world was observed in other scientific fields such as infectious diseases, environmental research [56], ophthalmology [57], and substance use [32]. Sweileh et al. investigated research activity on substance use in the Arab region and found that Saudi Arabia ranked first followed by Lebanon and Egypt with publications from Lebanon having the highest number of citations per article [32]. Meho et al. did a bibliometric analysis of mental health research in the Arab region [31] and concluded that in terms of quantity, Egypt ranked first followed by Saudi Arabia and Lebanon. However, in terms of quantity per capita, Lebanon ranked first followed by Qatar. In the current study, Egypt ranked third in terms of quantity while Jordan ranked second. One potential explanation for the advanced ranking of Jordan in this field is the active research in Jordan in the field of nursing. Jordan ranked first in nursing research followed by Saudi Arabia and Egypt [58]. Important fields in nursing include community health, mental health, and adolescent nursing research and this explain why the percentage of articles from the Arab region in the nursing subject area was higher than that at the global level. The researcher who ranked second was in the nursing field and based in Jordan.

The average number of citations of the retrieved articles was relatively low compared to articles in other fields [59, 60]. This suggests that there is a limited number of researchers interested in the field of mental health research among young adults. The findings that many Scopus-indexed journals in the Arab regions participated in publishing the retrieved articles could be another possible reason for the relatively low number of citations. Local journals in the Arab regions that are in the general medical or public health field might be less attractive compared to specialized and international journals in the field of mental health and well-being. Stress among medical students constituted a major theme in the retrieved articles. In the Arab region, the move from school education to university education is emotionally and mentally dramatic especially in the college of medicine and that is why a lot of research from the Arab region was about academic stress in medical students [61–63].

The current study was the first of its type to assess the research in the Arab region in the field of mental health and wellbeing in university students. However, Hernández-Torranoa et al. published a bibliometric analysis of global research on mental health and wellbeing in university students [20]. The authors used one database, namely the Web of Science, and retrieved 5561 documents for the study period from 1975 to 2020. In the current study, we retrieved 5554 articles at the global level during the study period from 2001 to 2020. When studying the global research activity, as in the case of Hernández-Torranoa et al., the research activity before 2000 was included because the global number of publications was high. However, in the case of the current study, the number of publications in the Arab region on the topic before 2001 was limited. Key findings of Hernández-Torranoa et al. study were that research on mental health and well-being in university students: (a) experienced a noticeable linear growth since 2010; (b) published in a diverse and large number of different journals; (c) was mainly produced in the United States; (d) has little research collaboration; and (e) addressed seven research topics including positive mental health, mental disorders, substance abuse, counseling, stigma, stress, and mental health measurement. There is a research gap in the topics discussed globally and those published in the Arab region. Again, this could be due to lack of experience, cultural and religious reasons, and inadequate and poor international research collaboration.

The current study has a few limitations. The use of Scopus to assess research on mental health and well-being of university students in the Arab region might underestimate the research activity because several journals in the Arab region are not indexed in Scopus. However, most journals in general medicine, public health, and psychiatry in the Arab region are indexed in Scopus and many have a moderate impact factor. Therefore, the author expected that the number of missing documents to be minimum. Furthermore, using two different databases to maximize the number of retrieved documents will not allow for mapping because data from different databases have different and non-compatible formats. A second limitation is potential mistakes in the ranking of institutions or authors due to different spelling in different journals. Therefore, the research volume on university students does not represent the true research activity on youth and adolescents in the Arab world.

## Conclusions

University students represent a powerful human asset for development in the Arab region. Research on mental health and well-being of this category in the Arab region is important given the life pressures and cultural constraints. The current study noticed (1) an increasing interest and research activity by researchers in the Arab region lately, and (2) certain research gaps in mental health research including substance use, eating disorders, suicide, adjustment disorders, self-esteem, and happiness. In response to the limited research output and research gaps in the field of mental health and well being the following action measures need to be taken: training in research skills, strengthening research collaboration, implementing research recommendations, supporting researchers to cover for publishing in highly reputable journals, and finally translating and validating different measuring scales used in mental health research to help researchers in the Arab region to conduct research. Research implemented to screening for mental health issues among university students is also recommended especially in countries with political and economic unrest. The author of the current study also recommends journal editors in the field of psychology, mental health, and psychiatry index the journals in international databases to increase readability and citations. The current study is one of the few that analyzed research publications on people at a critical age, who are under pressure to succeed academically and at the same time lead the social and political change in their countries. Future research needs to broaden the group and investigate the research activity on adolescent health in general and mental health in specific.

## Data Availability

all data presented in this manuscript are available on the Scopus database using the search query listed in the methodology section.

## References

[1] Lancaster P (2009). Youth: the great 21st-century challenge. Middle East.

[2] Waterbury J (2019). Reform of higher education in the Arab world. Major challenges facing higher education in the Arab World: Quality assurance and relevance.

[3] World Health Organization (WHO). Mental Health: Strengthening our response. https://www.who.int/news-room/fact-sheets/detail/mental-health-strengthening-our-response.

[4] Thomas SP (2013). World Health Assembly adopts comprehensive Mental Health Action Plan for 2013–2020. Issues Ment Health Nurs.

[5] Hemelaar J, Elangovan R, Yun J, Dickson-Tetteh L, Fleminger I, Kirtley S, Williams B, Gouws-Williams E, Ghys PD (2019). Alash’le GA. Global and regional molecular epidemiology of HIV-1, 1990–2015: a systematic review, global survey, and trend analysis. Lancet Infect Dis.

[6] de Martel C, Plummer M, Vignat J, Franceschi S (2017). Worldwide burden of cancer attributable to HPV by site, country and HPV type. Int J Cancer.

[7] Harb C. The Arab region: Cultures, values, and identity. 2016.

[8] Galal A, Kanaan T. Financing higher education in Arab countries. In: Economic Research Forum (ERF) Policy research report. 2010; 2010: 29–47.

[9] El-Araby A (2011). A comparative assessment of higher education financing in six Arab countries. Prospects.

[10] Mulderig MC. An uncertain future: Youth frustration and the Arab Spring. 2013.

[11] Samarah WA (2017). The Palestinian economy and the Arab spring. MERPA.

[12] Nassar H, Biltagy M (2017). Poverty, employment, investment, and education relationships: the case of Egypt. Sage Open.

[13] Backeberg L, Tholen J (2018). The frustrated generation youth exclusion in Arab Mediterranean societies. J Youth Stud.

[14] Kronfol Z, Khalifa B, Khoury B, Omar O, Daouk S, deWitt J, ElAzab N, Eisenberg D (2018). Selected psychiatric problems among college students in two Arab countries: comparison with the USA. BMC Psychiatry.

[15] Hinnebusch R (2015). Globalization, democratization, and the Arab uprising: the international factor in MENA’s failed democratization. Democratization.

[16] Fargues P, Fandrich C. Migration after the arab spring. 2012.

[17] Kovacheva S, Popivanov B, Kabaivanov S. Youth policy in Arab Mediterranean countries in a comparative perspective. SAHWA Policy Report. 2017;6:45.

[18] Kostenko VV, Kuzmuchev PA, Ponarin ED (2016). Attitudes towards gender equality and perception of democracy in the Arab world. Democratization.

[19] Roy O (2017). Political Islam After the Arab Spring: Between Jihad and Democracy. Foreign Aff.

[20] Hernández-Torrano D, Ibrayeva L, Sparks J, Lim N, Clementi A, Almukhambetova A, Nurtayev Y, Muratkyzy A (2020). Mental health and well-being of university students: A bibliometric mapping of the literature. Front Psychol.

[21] Harzing A-W, Alakangas S (2016). Google Scholar, Scopus and the Web of Science: a longitudinal and cross-disciplinary comparison. Scientometrics.

[22] Martín-Martín A, Orduna-Malea E, Thelwall M, López-Cózar ED (2018). Google Scholar, Web of Science, and Scopus: A systematic comparison of citations in 252 subject categories. Journal of informetrics.

[23] Zyoud SH (2016). Dengue research: a bibliometric analysis of worldwide and Arab publications during 1872–2015. Virol J.

[24] Aparicio-Martinez P, Perea-Moreno AJ, Martinez-Jimenez MP, Redel-Macías MD, Vaquero-Abellan M, Pagliari C (2019). A bibliometric analysis of the health field regarding social networks and young people. Int J Environ Res Public Health..

[25] Li S, Wang H, Zheng H, Li N, Sun C, Meng X, Zheng W, Wang K, Qin H, Gao W, et al. Bibliometric analysis of pediatric liver transplantation research in PubMed from 2014 to 2018. Med Sci Monit*. *2020; 26:e922517. 10.12659/MSM.922517 PMC7294844 32493895

[26] Sachs SE, Sachs JD (2007). Mental health in the millennium development goals: not ignored. PLoS Med.

[27] Vogel JP, Pileggi-Castro C, Chandra-Mouli V, Pileggi VN, Souza JP, Chou D, Say L (2015). Millennium Development Goal 5 and adolescents: looking back, moving forward. Arch Dis Childhood.

[28] Izutsu T, Tsutsumi A, Minas H, Thornicroft G, Patel V, Ito A (2015). Mental health and wellbeing in the sustainable development goals. Lancet Psychiatry.

[29] Al-Musawi NM (2001). Psychometric properties of the Beck Depression Inventory-II with University students in Bahrain. J Pers Assess.

[30] van Eck NJ, Waltman L (2010). Software survey: VOSviewer, a computer program for bibliometric mapping. Scientometrics.

[31] Zeinoun P, Akl EA, Maalouf FT, Meho LI (2020). The Arab Region’s Contribution to Global Mental Health Research (2009–2018): a bibliometric analysis. Front Psychiatry.

[32] Sweileh WM, Sa’ed HZ, Al-Jabi SW, Sawalha AF (2014). Substance use disorders in Arab countries: research activity and bibliometric analysis. Subst Abuse Treat Prev Policy.

[33] Massad SG, Shaheen M, Karam R, Brown R, Glick P, Linnemay S, Khammash U (2016). Substance use among Palestinian youth in the West Bank, Palestine: a qualitative investigation. BMC Public Health.

[34] Alhyas L, Al Ozaibi N, Elarabi H, El-Kashef A, Wanigaratne S, Almarzouqi A, Alhosani A, Al Ghaferi H (2015). Adolescents’ perception of substance use and factors influencing its use: a qualitative study in Abu Dhabi. JRSM Open.

[35] Alblooshi H, Hulse GK, El Kashef A, Al Hashmi H, Shawky M, Al Ghaferi H, Al Safar H, Tay GK (2016). The pattern of substance use disorder in the United Arab Emirates in 2015: results of a National Rehabilitation Centre cohort study. Subst Abuse Treat Prev Policy.

[36] Loffredo CA, Boulos DN, Saleh DaA, Jillson IA, Garas M, Loza N, Samuel P, Shaker YE, Ostrowski M-J, Amr S (2015). Substance use by Egyptian youth: current patterns and potential avenues for prevention. Subst Use Misuse.

[37] Al Gaferi H, Osman OT, Matheson C, Wanigaratne S, Bond C (2013). Substance misuse in Arabic countries: the need for published research. Int J Prevent Treat Subst Use Disord.

[38] Melisse B, de Beurs E, van Furth EF (2020). Eating disorders in the Arab world: a literature review. J Eating Disord.

[39] Saleh RN, Salameh RA, Yhya HH, Sweileh WM (2018). Disordered eating attitudes in female students of An-Najah National University: a cross-sectional study. J Eat Disord.

[40] Musaiger AO, Al-Mannai M, Tayyem R, Al-Lalla O, Ali EY, Kalam F, Benhamed MM, Saghir S, Halahleh I, Djoudi Z (2013). Risk of disordered eating attitudes among adolescents in seven Arab countries by gender and obesity: a cross-cultural study. Appetite.

[41] Martin H, Ammerman SD (2002). Adolescents with eating disorders. Primary care screening, identification, and early intervention. Nurs Clin North Am.

[42] Rowe E (2017). Early detection of eating disorders in general practice. Aust Fam Physician.

[43] D’raven LL, Pasha–Zaidi N (2015). Happiness in the United Arab Emirates: conceptualisations of happiness among Emirati and other Arab students. Int J Happiness Dev.

[44] Petkari E, Ortiz-Tallo M (2018). Towards youth happiness and mental health in the United Arab Emirates: The path of character strengths in a multicultural population. J Happiness Stud.

[45] Alansari B, AlAli T (2017). Validation of the Arabic version of the oxford happiness inventory among undergraduates in Kuwait. Eur Psychiatry.

[46] Aboalshamat K, Alsiyud A, Al–Sayed R, Alreddadi R, Faqiehi S, Almehmadi S (2018). The relationship between resilience, happiness, and life satisfaction in dental and medical students in Jeddah, Saudi Arabia. Niger J Clin Pract.

[47] D’raven LL, Pasha-Zaidi N (2014). Happiness strategies among Arab university students in the United Arab Emirates. Happiness Well-Being.

[48] Abdel-Khalek AM, Lester D (2017). The association between religiosity, generalized self-efficacy, mental health, and happiness in Arab college students. Personal Individ Differ.

[49] Francis LJ, Ok Ü, Robbins M (2017). Religion and happiness: a study among university students in Turkey. J Religion Health.

[50] Abdel-Khalek AM (2012). Associations between religiosity, mental health, and subjective well-being among Arabic samples from Egypt and Kuwait. Mental Health Religion Culture.

[51] Morad M, Merrick E, Schwarz A, Merrick J (2005). A review of suicide behavior among Arab adolescents. Sci World J.

[52] Mazaba ML, Mulenga D, Mwenya Kwangu B, Njunju EM, Siziya S (2017). Suicidal ideation among Arab adolescents in school in the west bank. Global View Suicid Ideat Adolesc.

[53] Baroud E, Ghandour LA, Alrojolah L, Zeinoun P, Maalouf FT (2019). Suicidality among Lebanese adolescents: prevalence, predictors and service utilization. Psychiatry Res.

[54] Hsieh N (2017). A global perspective on religious participation and suicide. J Health Soc Behav.

[55] Lim K-S, Wong CH, McIntyre RS, Wang J, Zhang Z, Tran BX, Tan W, Ho CS, Ho RC. Global lifetime and 12-month prevalence of suicidal behavior, deliberate self-harm and non-suicidal self-injury in children and adolescents between 1989 and 2018: a meta-analysis. Int J Environ Res Public Health 2019; 16(22):4581. 10.3390/ijerph16224581 PMC6888476 31752375

[56] Zyoud SH, Fuchs-Hanusch D, Zyoud S, Al-Rawajfeh A, Shaheen H (2017). A bibliometric-based evaluation on environmental research in the Arab world. Int J Environ Sci Technol.

[57] Sweileh WM, Al-Jabi SW, Shanti YI, Sawalha AF, Zyoud SH (2015). Contribution of Arab researchers to ophthalmology: a bibliometric and comparative analysis. Springerplus.

[58] Sweileh WM, Huijer HA, Al-Jabi SW, Zyoud SH, Sawalha AF (2019). Nursing and midwifery research activity in Arab countries from 1950 to 2017. BMC Health Serv Res.

[59] Sa’ed HZ, Al-Jabi SW, Sweileh WM (2015). Scientific publications from Arab world in leading journals of integrative and complementary medicine: a bibliometric analysis. BMC Complement Altern Med.

[60] Sweileh WM, Al-Jabi SW, Sa’ed HZ, Sawalha AF, Ghanim MA (2014). Osteoporosis is a neglected health priority in Arab World: a comparative bibliometric analysis. Springerplus.

[61] Masri R, Kadhum M, Farrell SM, Khamees Aa, Al-Taiar H, Molodynski A (2019). Wellbeing and mental health amongst medical students in Jordan: a descriptive study. Int Rev Psychiatry.

[62] Fawzy M, Hamed SA (2017). Prevalence of psychological stress, depression and anxiety among medical students in Egypt. Psychiatry Res.

[63] Gazzaz ZJ, Baig M, Al Alhendi BSM, Al Suliman MMO, Al Alhendi AS, Al-Grad MSH, Qurayshah MAA. Perceived stress, reasons for and sources of stress among medical students at Rabigh Medical College, King Abdulaziz University, Jeddah, Saudi Arabia. BMC Med Educ. 2018; 18(1):1–9. 10.1186/s12909-018-1133-2 PMC5824476 29471824

